# The impact of cold spells on mortality and effect modification by cold spell characteristics

**DOI:** 10.1038/srep38380

**Published:** 2016-12-06

**Authors:** Lijun Wang, Tao Liu, Mengjue Hu, Weilin Zeng, Yonghui Zhang, Shannon Rutherford, Hualiang Lin, Jianpeng Xiao, Peng Yin, Jiangmei Liu, Cordia Chu, Shilu Tong, Wenjun Ma, Maigeng Zhou

**Affiliations:** 1 The National Center for Chronic and Non-communicable Disease Control and Prevention, Chinese Center for Disease Control and Prevention, Beijing, China; 2 Guangdong Provincial Institute of Public Health, Guangdong Provincial Center for Disease Control and Prevention, Guangzhou, Guangdong, China; 3 Shanghai Minhang Center for Disease Control and Prevention, Shanghai, China; 4 Guangdong Provincial Center for Disease Control and Prevention, Guangzhou, Guangdong, China; 5 Center for Environment and Population Health, School of Environment, Griffith University, Brisbane, Queensland, Australia; 6 School of Public Health and Social Work, Institute of Health and Biomedical Innovation, Queensland University of Technology, Brisbane, Queensland, Australia

## Abstract

In China, the health impact of cold weather has received little attention, which limits our understanding of the health impacts of climate change. We collected daily mortality and meteorological data in 66 communities across China from 2006 to 2011. Within each community, we estimated the effect of cold spell exposure on mortality using a Distributed Lag Nonlinear Model (DLNM). We also examined the modification effect of cold spell characteristics (intensity, duration, and timing) and individual-specific factors (causes of death, age, gender and education). Meta-analysis method was finally used to estimate the overall effects. The overall cumulative excess risk (CER) of non-accidental mortality during cold spell days was 28.2% (95% CI: 21.4%, 35.3%) compared with non-cold spell days. There was a significant increase in mortality when the cold spell duration and intensity increased or occurred earlier in the season. Cold spell effects and effect modification by cold spell characteristics were more pronounced in south China. The elderly, people with low education level and those with respiratory diseases were generally more vulnerable to cold spells. Cold spells statistically significantly increase mortality risk in China, with greater effects in southern China. This effect is modified by cold spell characteristics and individual-level factors.

Mortality is well known to be associated with extreme meteorological conditions, including heat waves and cold spells^1^,^2^. Many studies have examined the relationship between extreme temperature events and mortality^3^,^4^,^5^,^6^,^7^,^8^, though cold-related mortality is much less studied^9^,^10^,^11^,^12^,^13^. Increases in mortality with decreasing temperatures in winter have been mostly reported in mid-latitude developed countries^12^,^14^,^15^,^16^. In contrast to heat wave effects, which appear to last several days, the effects of cold spells may persist for up to several weeks^17^. Even though global warming will substantially increase health impacts of heat wave episodes, previous studies reported that cold-related mortality is still an important public health issue, particularly in temperate regions and should not be underestimated by public health authorities. In China, the health impact of cold weather has received little attention, and only a few studies have examined the health effects of cold spells and these were mostly focused on a single or few cities^9^,^18^,^19^,^20^.

Although the Intergovernmental Panel on Climate Change projects that there will be more frequent hot and fewer cold temperature extremes over most land areas as global mean temperatures increase, occasional cold winter extremes will continue to occur^21^, which could increase the risk of cardio-respiratory diseases and premature deaths, particularly in vulnerable populations with limited adaptation resources^9^. The relationships between cold spells and mortality can be modified by many factors, including socio-demographic status, housing, heating equipment and cold spell characteristics. Although previous studies reported that the intensity, duration and timing of heat waves could modify the effects of extreme heat events on mortality^6^, little is known about which characteristics of cold spells, if any, are most relevant to public health.

China, located in East Asia, is a vast country with the largest population in the world. It is divided into 7 geographical areas with different types of climate, including tropical, subtropical, temperate and plateau mountain zones. As climate change has different effects on the geographically diverse regions, changes in extreme events show large regional variations^22^. Recently, China has experienced frequent and intense cold spells, especially in 2008 when one cold spell seriously affected 20 provinces in south China, resulting in substantial excess mortality^23^. These findings suggest that it is necessary to assess the impact of cold spells on mortality and the ways cold spell characteristics modify these effects across China.

This study investigated the effects of cold spells on mortality, and explored whether cold spell characteristics modified these effects in China using advanced time-series methods. The findings of this study will improve our understanding of the relationships between extreme cold events and mortality in geographically diverse regions, and provide evidence to support the development of effective adaptation strategies to mitigate the adverse effects of extreme cold events in the future.

## Methods

### Study settings

In the current study we identified 66 communities from 7 geographical regions of China, which constitute a total population of around 43 million^24^. All communities where the population size was over 200,000 were included in the current study to ensure sufficient daily death counts for every surveillance point in model fitting for time series analysis. The details of the study settings are described in supplementary material Fig. S1 and Table S1.

### Data collection

Daily non-accidental mortality data from the cold season (November to March) in 2006 to 2011 were obtained from the Chinese Center for Disease Control and Prevention. The original source of mortality data were death certificates, which included the causes and dates of death, gender and age. Causes of death were categorized using the International Classification of Diseases 10^th^ Revision (ICD–10 codes: A00–R99 for non-accidental diseases)^23^. The codes J00–J99 represent respiratory diseases (RESP), the codes I00-I99 represent cardiovascular diseases (CVD), and the codes I60-I69 represent cerebrovascular diseases (CBD). We also divided daily deaths into several strata by gender, age groups (0–64 years, 65–74 years 75–84 years and 85 years or older) and education attainment. The education attainment levels were classified into three levels: primary school for less than 6 years of schooling, middle school for 6–9 school years, and college or higher for 9 years or more.

The corresponding community-specific daily meteorological data were collected from the China Meteorological Data Sharing Service System network, which is a compilation of quality-controlled national surface observations in China (http://www.escience.gov.cn/metdata/page/index.html). Meteorological data for each community was obtained from one basic-reference surface weather observation station or automatic station located in the community. If there was no weather station location in the community, the data from the nearest weather station was collected. Meteorological data included daily average temperature (TM), minimum temperature (TMin), maximum temperature (TMax), relative humidity (RH) and wind speed (WS).

### Definitions and statistical analysis

#### Definition of cold spell and cold spell characteristics

Various approaches have been used to define a cold spell across different studies^10^,^11^,^25^,^26^. For instance, Kysely *et al*. defined a cold spell as “a period of days on which air temperature did not exceed −3.5 °C”^10^, and Hickey suggested that “a cold spell could consist of a period of 10 consecutive days when the minimum air temperature was 5 °C or more below normal”^26^. However, using a unique temperature as a threshold may not be appropriate in a spatially large country like China, and it has been suggested to use the percentile of daily temperature to define a cold spell^9^. In this study, we defined a weather fluctuation as a cold spell if the mean daily temperature fell below the 5^th^ percentile of the study period (cold season in 2006–2011) in a specific community for at least 2 consecutive days. We used the mean temperature to define a cold spell because it reflects the exposure throughout the whole day, while minimum or maximum temperature only represent a short period. In addition, daily average temperature can be more easily interpreted for decision making purposes^19^,^27^. Following the method used in a previous study^28^,^29^, the cold spell is defined as a binary variable assuming a value of 1 during the cold spell period.

The effect was estimated as the excess risk (ER) in daily mortality during cold spell days compared with non-cold spell days. To estimate the effect modification by cold spell characteristics, we characterized cold spells by their duration, intensity and timing within the season. Cold spell duration was measured using each event’s length in days, which is zero on the first day of the cold spell, one on the second day, two on the third day, and so on. It was categorized into three levels according to the number of consecutive cold spell days: 2 days (short duration), 3 to 5 days (moderate duration), and 6 days or more (long duration). Cold spell intensity was measured using different cut-off points of cold spell thresholds: the 5^th^ and 2.5^th^ percentiles of the study period, community-specific daily mean temperature distribution. Cold spell timing was measured using the difference in days between the onset of the cold spell and the start of the cold season which is defined as 1^st^ November (this variable is zero on non-cold spell days).

#### Statistical analysis

We adopted a Poisson regression with a Distributed Lag Nonlinear Model (DLNM) to estimate the relationship between cold spell and mortality for each community^30^. The Akaike Information Criterion was used to judge the optimal degrees of freedom (*df*). We used the following Poisson regression model in each community:


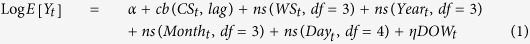


where *t* is the day of observation; *E* [*Y*_*t*_] is the number of deaths on day *t; α* is intercept; *CS* represents the cold spell on day *t* (0 = non-cold spell days, and 1 = cold spell days); *cb* means “cross-basis” function, in which a linear function and a natural cubic spline function were used to estimate the linear and lagged effect of cold spell, respectively. To completely capture the overall effects of cold spell exposure, a lag structure of up to 27 days was fitted, which is consistent with previous studies^9^,^23^. Here, *CS* was separately defined by two thresholds of mean daily temperature (5^th^ and 2.5^th^ percentiles) that were used to represent different cold spell intensities; *ns* means the natural cubic spline function; *WS*_*t*_ refers to the daily average wind speed with *3 df*s; Another *3 dfs* were used to smooth year, calendar month and day to control for secular and seasonal trends; *DOW* is a dummy variable representing day of the week, and η is vector of coefficients. In this model, ER were reported, which indicated the percentage of increased death risk due to exposure to a cold spell compared to the non-cold spell. The cumulative effect of cold spell on mortality during the lag 0–27 days was defined as cumulative excess risk (CER).

In order to estimate the modifying effect of cold spell duration, we replaced the model [1] with


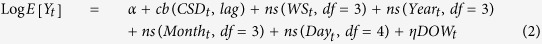


where *CSD* represents the cold spell duration (0 = non-cold spell days, 1 = cold spell lasting 2 days, 2 = cold spell lasting 3–5 days, and 3 = cold spell lasting 6 or more days), and *cb* stands for “cross-basis” function, in which a B-spline function and a natural cubic spline function were used to estimate the non-linear and 27 days lagged effect of cold spell, respectively. “Non-cold spell days” were defined as the reference group for calculating ER. Other parameter settings were the same as in model [1]. The cumulative excess mortality risks during the lag 0–27 days for different cold spell durations were calculated.

The model [1] was also used to estimate the modifying effects of cold spell timing, in which the variable *CS* was replaced with *CST* (cold spell timing). In the “cross-basis” function, a linear function and a natural cubic spline function were used to estimate the linear and 27 days lagged effect of cold spell timing, respectively. The cumulative excess mortality risks during the lag 0–27 days for every 10 days earlier of cold spell onset were calculated.

We further assessed the modifying effects of individual characteristics including causes of death, age group, gender and education. We employed the model [1] to separately estimate the CER of cold spell (defined by the 5^th^ percentiles of air temperature) on mortality in individuals with different causes of death (total non-accidental mortality, CVD, CBD and RESP), death ages (0–64 years, 65–74 years, 75–84 years and 85 years or older), gender (males and females) and education attainment (primary school or lower, middle school and college or higher).

All above analyses were conducted in each community, and then a meta-analysis model was used to estimate the summary effects of cold spells on mortality in all study communities and different regions. The combined CER of the cold effect among different communities was defined as summary CER. A multivariate meta-analysis model was performed to estimate the summary lag structure of cold effects^9^,^23^,^31^.

### Sensitivity analysis

Sensitivity analyses were carried out by changing the *df* for the temporal spline with *2–4 df* per year and *2–4 df* per calendar month in the DLM, in order to test the robustness of results.

The study was approved by Chinese Center for Disease Control and Prevention. As aggregated data with no personal information were involved. All statistical tests were two-sided and values of p < 0.05 were considered statistically significant. The results are expressed as CER (%) of deaths during cold spells with a delay of 27 days. We used the “dlnm” package in R software for the Poisson regression analysis. Traditional meta-analysis and multivariate meta-analysis were conducted using the “metafor” package and “mvmeta” package, respectively.

## Results

### General characteristics

Table 1 shows the distributions of mortality, temperature and relative humidity during the cold spell and non-cold spell days in 66 communities. The average cold spell length was 2.77 days across all studied communities. The average cold spell days generally increased from 2006 to 2010, and slightly decreased from 2010 to 2011 (Fig. S2). Most of the cold spells occurred in January (Fig. S3). The average number of daily deaths was 9.6 across all communities, and a total of 63,966 non-accidental deaths were recorded during the cold spell days. Compared with the non-cold spell days, cold spell days had a lower temperature and relative humidity, and higher mortality. Detailed information for each included community is shown in Table S1.

### Effects of cold spell on mortality

**Figure 1:**
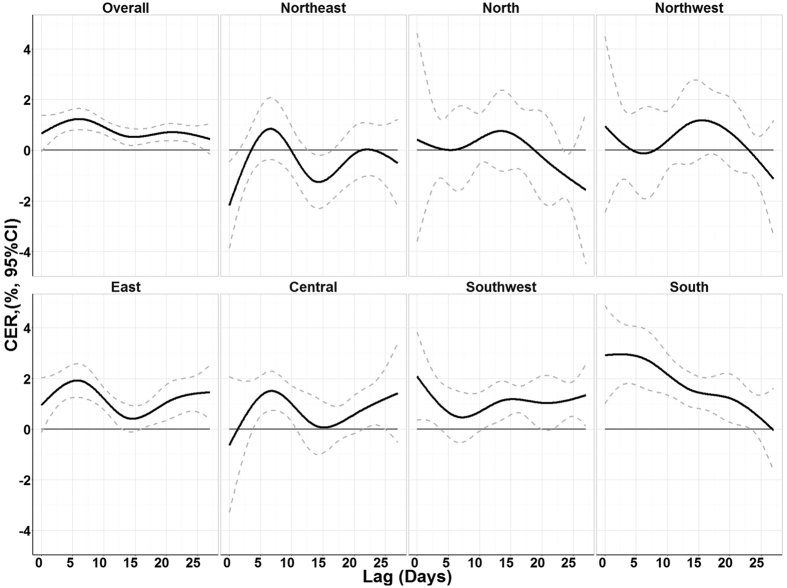
Summary single day CER (%, 95% CI) of cold spells on total non-accidental mortality during lag 0–27 days in different regions in China, 2006–2011. CER: Cumulative excess risk of mortality for cold spell exposure during lag 0–27 days. A cold spell was defined as a weather fluctuation if the mean daily temperature fell below the 5th percentile of the study period (cold season in 2006–2011) in a specific community for at least 2 consecutive days.

Figure 1 illustrates the lag structure of associations between cold spell and mortality by region. The mortality risk reached a maximum after 5 days exposure to a cold spell, then decreased and persisted for the next 3 weeks. This pattern was observed in most geographical regions.

**Figure 2:**
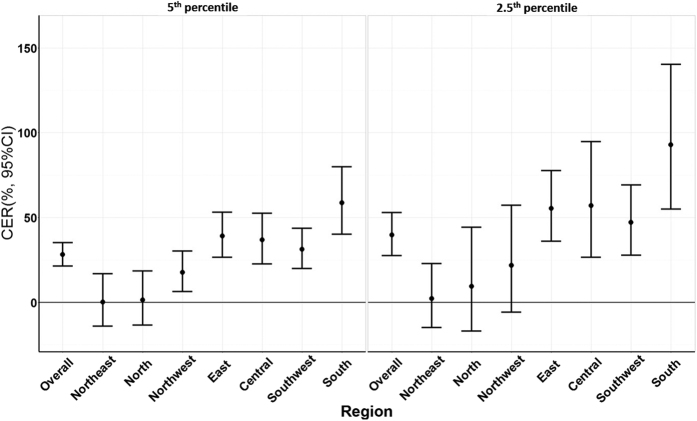
Summary CER (95% CI) of different intensity of cold spells on total non-accidental mortality during lag 0–27 days in different regions of China, 2006–2011. CER: Cumulative excess risk of mortality for cold spell exposure during lag 0–27 days. In the left plot, a cold spell was defined as a weather fluctuation if the mean daily temperature fell below the 5th percentile of the study period (cold season in 2006–2011) in a specific community for at least 2 consecutive days. In the right plot, a cold spell was defined as a weather fluctuation if the mean daily temperature fell below the 2.5th percentile of the study period (cold season in 2006–2011) in a specific community for at least 2 consecutive days.

Figure 2 provides a summary of CERs of cold spells for the 2 different intensity categories on mortality during lag 0–27 days for the 7 regions. Compared with non-cold spell days, the CER on mortality was 28.5% (95% CI: 21.4%, 35.3%) for the exposure to cold spell with the 5^th^ percentile as the intensity threshold, and was 39.7% (95% CI: 27.6%, 52.9%) for cold spell with the 2.5^th^ percentile as the threshold. In terms of regional distribution, the maximum overall effect was observed in south China (CER = 58.7% and 92.9% for cold spells with the 5^th^ and 2.5^th^ percentiles as threshold cut-offs, respectively), followed by east China (CER = 39.2% and 55.5%) and central China (CER = 36.8% and 57.1%). We did not find statistically significant effects of cold spells on mortality in the northeast (CER = 0.2% and 2.3%) or north China (CER = 1.4% and 9.5%). Figure S4 indicates the community-specific RR (95% CI) of cold spells on total non-accidental mortality during lag 0–27 days in 66 communities.

### Effect modification by cold spell characteristics

Region specific analyses showed that the number of daily deaths varied by region, but increased with more intense cold spells. South China had the highest average daily mean temperature (8.38 °C) and northeast China had the lowest average daily mean temperature (−17.42 °C). Northeast China had the highest average intensity (−20.22 °C) and south China had the lowest average intensity (1.45 °C). Most of the cold spells occurred in central China and the longest average duration cold spell was in northeast China (4.60 days). More detailed information is shown in Table S3.

**Figure 3:**
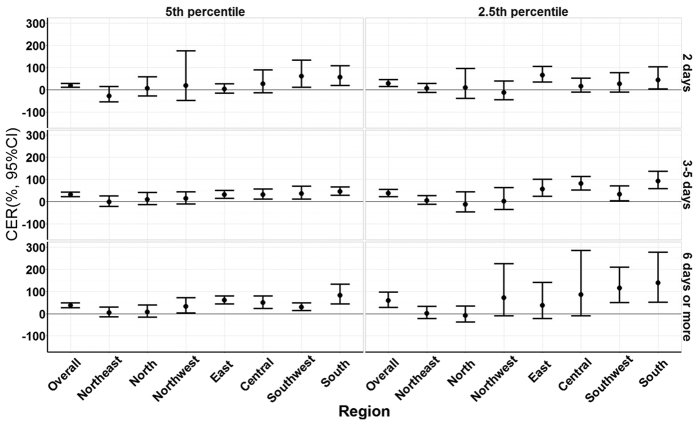
Summary CER (95% CI) of cold spells with different intensity and duration on total non-accidental mortality during lag 0–27 days in different regions of China, 2006–2011. CER: Cumulative excess risk of mortality for cold spell exposure during lag 0–27 days. In the left plots, a cold spell was defined as a weather fluctuation if the mean daily temperature fell below the 5th percentile of the study period (cold season in 2006–2011) in a specific community for at least 2 consecutive days. In the right plots, a cold spell was defined as a weather fluctuation if the mean daily temperature fell below the 2.5th percentile of the study period (cold season in 2006–2011) in a specific community for at least 2 consecutive days. Under each definition of a cold spell, the duration of cold spells were divided into three groups: short duration (lasting 2 days), moderate duration (lasting 3 to 5 days) and long duration (lasting 6 days or more).

Figure 3 shows the CERs of cold spells with different intensities and different durations on non-accidental mortality during lag 0–27 days by region. An increase in mortality when cold spell duration and intensity increased was observed for most regions. For example, when the intensity was defined by the 5^th^ percentile cut-off, the CERs increased from 18.9%, 31.9% to 36.3% for cold spells lasting 2, 3–5 and ≥6 days, and when the intensity was defined by a 1^st^ percentile cut-off, the CERs were 28.2%, 37.7% and 58.6% for cold spells lasting 2, 3–5 and ≥6 days, respectively. While this pattern was consistent in all 7 regions of China, not all results were statistically significant (detailed information is shown in Table S4).

**Figure 4:**
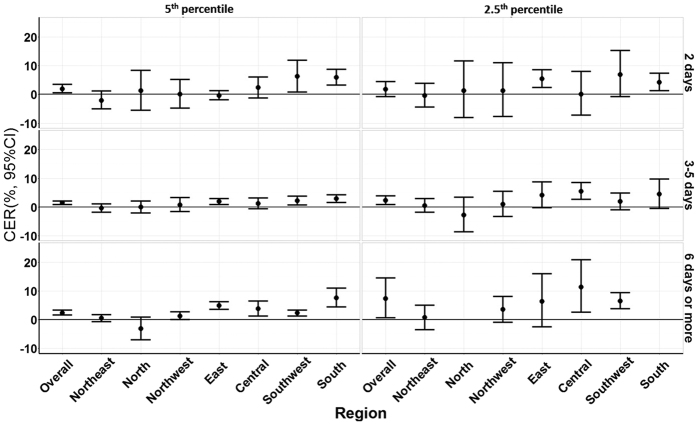
Summary CER (95% CI) of average 10 days earlier occurrence of cold spells on non-accidental mortality during lag 0–27 days in different regions of China, 2006–2011. CER: Cumulative excess risk of mortality for cold spell exposure during lag 0–27 days. In the left plots, a cold spell was defined as a weather fluctuation if the mean daily temperature fell below the 5th percentile of the study period (cold season in 2006–2011) in a specific community for at least 2 consecutive days. In the right plots, a cold spell was defined as a weather fluctuation if the mean daily temperature fell below the 2.5th percentile of the study period (cold season in 2006–2011) in a specific community for at least 2 consecutive days. Under each definition of a cold spell, the duration of cold spells were divided into three groups: short duration (lasting 2 days), moderate duration (lasting 3 to 5 days) and long duration (lasting 6 days or more). In the north and south communities, the effects were not estimated because there were very few cold spells lasting 6 days or more and the regression models failed to successfully estimate the effects of cold spell timing on mortality risk.

The CER of a cold spell also increased when the cold spell occurred early in the season, and this effect was strengthened as the cold spell became more intense and longer (Fig. 4). For example, when cold spell intensity was defined by the 5^th^ percentile cut-off and the durations increased from 2, 3–5 to ≥6 days, the average CERs on mortality respectively increased by 1.9% (95% CI: 0.5%, 3.4%), 1.4% (95% CI: 0.8%, 2.0%) and 2.4% (95% CI: 1.6%, 3.3%) for each 10 days earlier occurrence of cold spells. For the cold spells defined by the 1^st^ percentile cut-off, the corresponding average CERs increased by 1.8% (95% CI: −0.8%, 4.4%), 2.3% (95% CI: 0.8%, 3.9%) and 7.3% (95% CI: 0.6%, 14.5%), respectively. This phenomenon was also observed in regional analysis, with the effect found to be greater in southern than in northern China, but not all the results were statistically significant (the detailed information is shown in Table S5).

### Effect modification by individual factors

**Figure 5:**
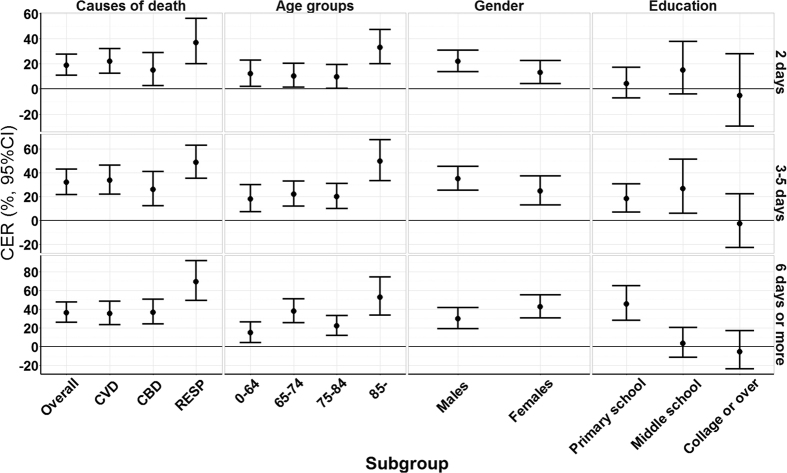
Summary CER (95% CI) of cold spells with different duration on total non-accidental mortality during lag 0–27 days in China, by age, gender, education, and causes of death. CER: Cumulative excess risk of mortality for cold spell exposure during lag 0–27 days. A cold spell in this figure was defined as a weather fluctuation if the mean daily temperature fell below the 5th percentile of the study period (cold season in 2006–2011) in a specific community for at least 2 consecutive days. The duration of cold spells were divided into three groups: short duration (lasting 2 days), moderate duration (lasting 3 to 5 days) and long duration (lasting 6 days or more).

Figure 5 displays the CERs of cold spells on total non-accidental mortality during lag 0–27 days by causes of death, age, gender and education. The estimated CERs on mortality were higher for people with RESP than those with CVD and CBD, for the elderly than the younger population, and for the less-educated than the highly educated. This pattern was similar for cold spells with different durations. In particular, we observed a slightly lower CER for mortality in females than in males for cold spells with short and moderate durations, but higher CERs in long duration cold spells. Detailed information is shown in Table S6.

### Sensitivity analysis

We changed the *df* of smoothness for year and calendar month in the model, which gave similar results (Table S7), suggesting our results are robust.

## Discussion

Global climate change is projected to increase the frequency, intensity and duration of extreme climate events^32^. In this study, we estimated the mortality effects of cold spells and the modification effects of cold spell and individual characteristics in China. To the best of our knowledge, this is the first nationwide comprehensive study using a multi-community approach in China to investigate the health impacts of cold spells.

Our study found that cold spells significantly increased mortality risk in China. The cumulative excess risk for the cold spells defined by the 5^th^ percentile cutoff was 28.2% (95% CI: 21.4%, 35.3%) after controlling for potential confounding factors. The underlying mechanisms for the observed excess mortality during cold spell days may be related to higher prevalence of respiratory infection during cold days, particularly influenza epidemics, and an increase in plasma cholesterol and plasma fibrinogen with low temperatures, coupled with a higher blood pressure in cold weather, which could lead to thrombosis through haemoconcentration and trigger an acute mortality event^33^. Moreover, cold spell exposure might have also contributed to the excess mortality by reducing access to health services^33^.

We further observed that the maximum effect was found to appear after 5 days’ exposure and lasted for around 4 weeks. This result is consistent with some previous studies^34^,^35^,^36^. In most regions, a second peak effect was observed, possibly attributed to the mortality displacement effect. In other words, after 5 days’ exposure to a cold spell, people with underlying diseases may die in advance, and due to the long delayed effect of the cold spells, more people may die, leading to a second mortality peak^35^. This finding suggests that we should not only pay attention to sudden effects of cold spell events, but also consider the delayed effects of cold spells when assessing the health effects and responding to cold spells. Otherwise, the effect may be underestimated.

The cold spell effect identified in this study was quantitatively different from that reported by previous studies^1^,^2^,^10^,^37^. For example, Kysely *et al*. observed a 6.3% increase in mortality risk in males for cold spell exposure in the Czech Republic^10^, and Huynen *et al*. found that the average excess mortality during the cold spells in the Dutch was 12.8%^1^. This might be related to the different definitions of cold spell and statistical approaches employed in some previous studies. Kysely *et al*. defined a cold spell as “a period of days on which air temperature did not exceed −3.5 °C^10^. In Huynen *et al*.’s study, they defined a cold spell as a period of at least 9 days with a minimum temperature of −5 °C or lower, of which at least 6 days have a minimum temperature of −10 °C or lower^1^. Moreover, the intensity and duration of cold spells also varied greatly in other reported studies^10^,^37^.

We found the cold spell effects on mortality varied with geography. The largest effect was observed in south China, followed by east China and central China, while non-statistically significant effects were observed in northeast and north China. This finding is consistent with some previous studies^12^,^38^,^39^ that show a greater cold effect in more southern regions in Europe, eastern United States and Eastern Asia. One possible reason for this is that populations in warm regions have a lack of physiological and behavioral acclimatization and tend to be most vulnerable to cold^38^. In addition, household and community adaptation measures such as indoor heating vary geographically, with fewer such measures in southern China than northern China. Another possible reason is that health care systems, especially emergency services in these regions are not prepared for the sudden growth for care as a result of cold spells. For example, during the extreme cold event in southern China, the number of ambulance calls rose to such an extent that nearly a quarter received no response^40^.

Intuitively, intensity, duration and timing of cold spells are important modifying factors of the cold-mortality relationship^21^. Similar to the modification effects of intensity, duration and timing of heat waves^5^,^41^, the current study found that the characteristics of cold spells might have also played an important role in the mortality risk of cold spells in China. Similar findings have also been reported in previous studies^18^,^37^,^42^. In our study, we found that there was a significant increase in non-accidental mortality for longer, stronger and earlier cold spells. The possible mechanism may point to the human body’s response via thermal regulation. When the ambient temperature is low, vasoconstriction of the extremities occurs to make sure the body drives oxygenated blood to the core to support vital organs. As the cold persists and heat is lost on the periphery via radiation the temperature gap between the core and the periphery increases. Heat is then redistributed to the periphery, which reduces core temperatures. Shivering, muscle contractions, an increased heart rate, and rapid breathing occur, in an effort to produce heat^43^,^44^. The lower the temperature or the longer the cold spell, the more work is required for the body’s system to maintain a normal temperature, therefore, more intense or longer cold spells are likely to have greater health effects. In terms of the mortality effect of cold spells occurring earlier in the season this could relate to the body needing time to adapt to environmental temperature change, and if the temperature drop is sharp and sudden earlier in the season, the body will not respond in time. These findings suggest that cold spell characteristics significantly modify the effect of cold spells, and indicate the importance of developing cold spell response plans for early, intense and long extreme cold events.

We also found that the cold spell effect on mortality increased with age. For example, the elderly aged >85 years were particularly susceptible to cold spell impacts. This finding is in accordance with some previous studies^2^,^12^,^20^. The elderly are likely to be more sensitive to cold spells because, in general, their health status is more compromised than younger people, which may leave them with a reduced thermoregulatory response and less sensitive thermal perception^1^. In addition, we found females are more vulnerable than males in longer duration cold spells, which is consistent with some previous studies^8^,^14^,^45^,^46^,^47^, and may be attributable to socioeconomic factors such as the high proportion of women among the elderly due to the longer expectancy life for women in China^46^. We further observed larger effects on mortality from respiratory diseases. This corresponds with other studies^2^,^12^. The possible reason may be due to increased infection from indoor crowding, the adverse effects of cold weather on the immune system, and the fact that low temperatures may facilitate the survival of bacteria and virus in droplets^48^,^49^. Education attainment is another important modifying factor of cold spell effects in our study, which may be related to low income, poor living, nutrition conditions and poor access to domestic heating and health facilities^50^,^51^. However, a study conducted in Shanghai found no statistically significant association between education and cold-mortality^52^. These individual level findings indicate adaptation strategies and measures should pay more attention to these vulnerable populations.

This study has several limitations. The confounding effect of air pollution is not considered because relevant data were not available. Although previous studies have found that the effect of air pollution was much smaller than that of temperature^53^, more studies are needed in the future to assess the potential confounding effects of ambient air pollutants on the cold effects on mortality at the national level in China. Another limitation is that data on incidence of acute respiratory infections were unavailable for this analysis. Acute respiratory infections such as influenza may have a strong influence on excessive deaths in winter^10^. However, our previous study conducted in Guangdong Province indicated that the association between the 2008 cold spell and mortality was not significantly modified by an influenza epidemic^9^.

## Conclusions

We found that cold spells significantly increased mortality risk in China, with a greater effect in southern areas. This effect was modified by cold spell characteristics and individual-specific factors. These findings suggest that adaptation measures to extreme cold events targeting vulnerable populations should be based on local climate patterns and socio-demographic status, and consider the varying cold spell characteristics in response mechanisms such as in the design and roll-out of health promotion campaigns.

## Additional Information

**How to cite this article**: Wang, L. *et al*. The impact of cold spells on mortality and effect modification by cold spell characteristics. *Sci. Rep.* **6**, 38380; doi: 10.1038/srep38380 (2016).

**Publisher's note:** Springer Nature remains neutral with regard to jurisdictional claims in published maps and institutional affiliations.

## Supplementary Material

Supplementary Information

## Figures and Tables

**Table 1:** Summary statistics of variables considered in this study.

	Mean	Min	25^th^	75^th^	Max
All days					
Non-accidental mortality	9.63	0	4.0	13.0	99.0
Mean temperature (°C)	4.88	−30.60	−0.30	10.90	27.30
Minimum temperature (°C)	1.01	−37.10	−4.50	7.50	25.20
Maximum temperature (°C)	9.98	−25.60	4.40	16.10	35.0
Relative humidity (%)	66.12	5.0	53.0	79.0	100.0
Cold spell days‡					
Non-accidental mortality	10.78	0	5.0	15.0	99.0
Mean temperature (°C)	−3.11	−30.60	−8.60	2.70	12.70
Minimum temperature (°C)	−6.29	−37.1	−12.7	0.60	12.1
Maximum temperature (°C)	1.26	−25.6	−3.1	6.40	23.2
Relative humidity (%)	64.38	7.0	51.0	79.0	100.0
Days per cold spell	2.77	2.00	5.50	12.50	16.00
Non-cold spell days					
Non-accidental mortality	9.50	0	4.0	13.0	94.0
Mean temperature (°C)	5.76	−25.60	0.70	11.50	27.30
Minimum temperature (°C)	1.81	−30.0	−3.7	8.0	25.2
Maximum temperature (°C)	10.94	−21.0	5.6	16.8	35.0
Relative humidity (%)	66.31	5.0	53.0	79.0	90.0

^‡^A cold spell was defined as a weather fluctuation if the mean daily temperature fell below the 5th percentile of the study period (cold season in 2006–2011) in a specific community for at least 2 consecutive days.
