# Supplementary material for: The impact of cold spells on mortality and effect modification by cold spell characteristics
Source: Sci Rep. 2016 Dec 6;6:38380. doi: 10.1038/srep38380 (PMC5138587; doi:10.1038/srep38380)
Supplement: Supplementary Information [file srep38380-s1.doc]

**Supplemental Material**

**The impact of cold spells on mortality and effect modification by cold spell characteristics**

Lijun Wang^1§^, Tao Liu^2§^, Mengjue Hu^2,3^, Weilin Zeng^2^,Yonghui Zhang^4^, Shannon Rutherford^5^, Hualiang Lin^2^, Jianpeng Xiao^2^, Peng Yin^1^, Jiangmei Liu^1^, Cordia Chu^5^ , Shilu Tong^6^, Wenjun Ma^2*^ , Maigeng Zhou^1 *^

*1. The National Center for Chronic and Non-communicable Disease Control and Prevention, Chinese Center for Disease Control and Prevention, Beijing, China*

*2. Guangdong Provincial Institute of Public Health, Guangdong Provincial Center for Disease Control and Prevention, Guangzhou, Guangdong, China*

*3. Shanghai Minhang Center for Disease Control and Prevention, Shanghai, China*

*4. Guangdong Provincial Center for Disease Control and Prevention, Guangzhou, Guangdong, China*

*5. Center for Environment and Population Health, School of Environment, Griffith University, Brisbane, Queensland, Australia*

*6. School of Public Health and Social Work, Institute of Health and Biomedical Innovation,* *Queensland University of Technology, Brisbane, Queensland, Australia*

§ These authors contributed equally to this work.

* To whom correspondence should be addressed.

**Corresponding author:**

Maigeng Zhou, The National Center for Chronic and Noncommunicable Disease Control and Prevention, Beijing, 100050, China

Tel: (8610)63041471, Fax: (8610)63041471, Mobile: 13611209306

Email: maigengzhou@126.com

Wenjun Ma, Guangdong Provincial Institute of Public Health, Guangdong Provincial Center for Disease Control and Prevention, Guangzhou, Guangdong, China

Tel: (8620)31051602, Mobile: 13622280436

Email: mawj@gdiph.org.cn

**
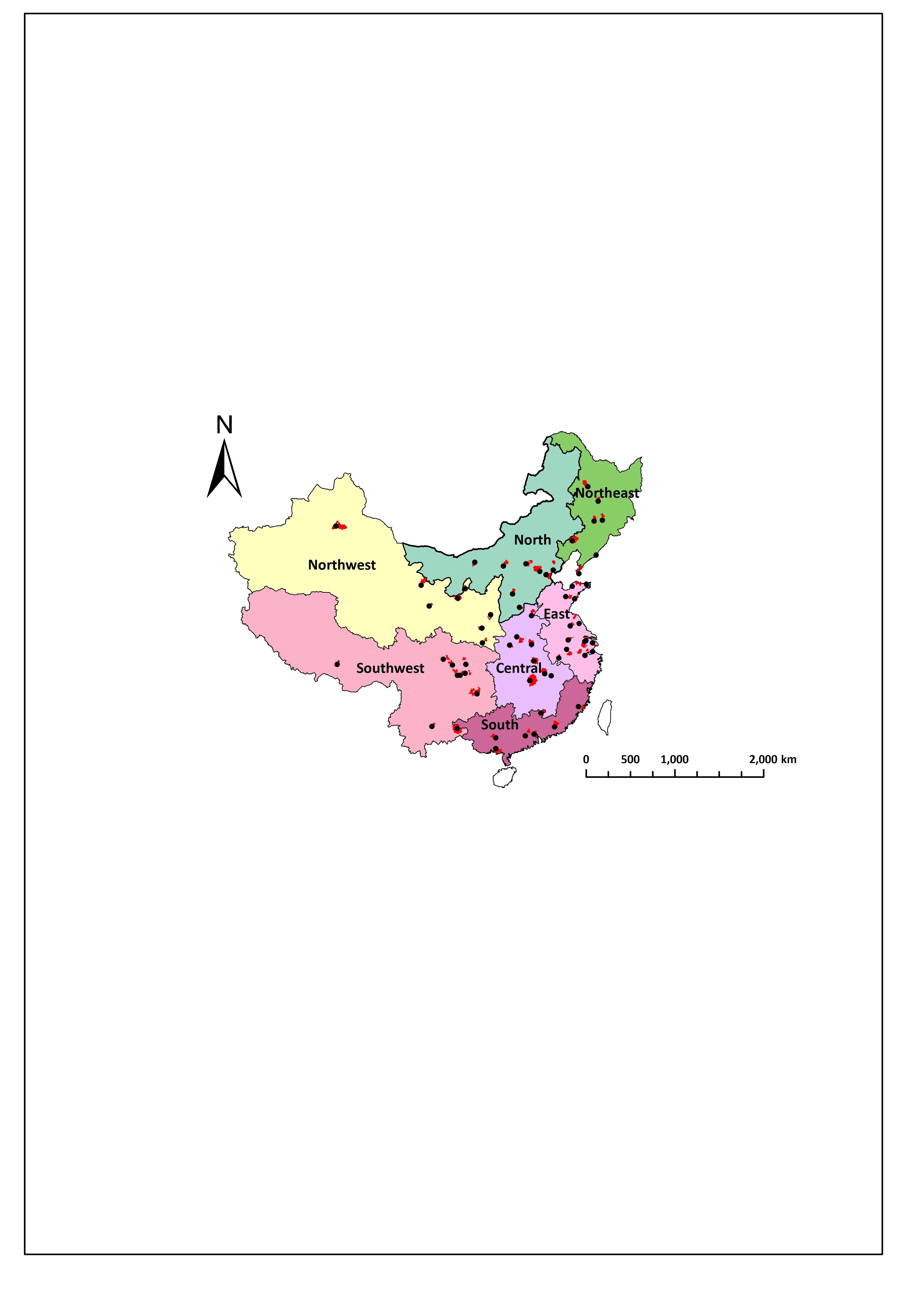
**

sFig 1. Distribution of 66 Chinese communities and meteorological stations included in the present study

Black dot: the position of meteorological stations; Red area: the position of included communities. This figure depicts the spatial distribution of 66 communities across China through ArcGIS [Version 9.3]. The map data were obtained from the National Geomatics Center of China (http://ngcc.sbsm.gov.cn/)

**sTable 1. The characteristics for 66 communities included in this study**

| City code | Region | Population | Latitude  (degrees N) | Age^+^65  (%) | No High  School (%) | Pop. density  (person/km^2^) | daily average deaths | Temperature (℃) | | | | | | | |
| --- | --- | --- | --- | --- | --- | --- | --- | --- | --- | --- | --- | --- | --- | --- | --- |
|  |  |  |  |  |  |  |  | mean | 1% | 5% | 25% | 50% | 75% | 95% | 99% |
| 210204 | Northeast | 480035 | 38.54 | 12.07 | 37.85 | 13910.1 | 10 | 11.3 | -9.7 | -5.9 | 2.7 | 12.8 | 20.8 | 24.9 | 26.6 |
| 210682 | Northeast | 543933 | 40.03 | 10.48 | 84.85 | 98.7 | 8 | 9.5 | -12.8 | -8.8 | -0.1 | 10.9 | 19.8 | 24.3 | 26.0 |
| 210921 | Northeast | 291609 | 42.04 | 8.48 | 86.82 | 106.9 | 10 | 8.3 | -16.8 | -12.7 | -3.9 | 9.8 | 20.9 | 25.4 | 27.2 |
| 220102 | Northeast | 390128 | 43.54 | 7.76 | 36.81 | 10962.0 | 6 | 6.5 | -21.4 | -17.2 | -6.3 | 8.5 | 19.7 | 25.1 | 26.6 |
| 220211 | Northeast | 643584 | 43.42 | 9.47 | 53.25 | 278.4 | 3 | 5.8 | -23.2 | -18.1 | -6.2 | 7.6 | 18.7 | 24.3 | 25.9 |
| 230103 | Northeast | 531526 | 45.45 | 8.76 | 32.87 | 22397.6 | 16 | 5.6 | -22.9 | -18.8 | -8.2 | 7.6 | 19.7 | 25.4 | 27.4 |
| 230223 | Northeast | 573180 | 47.23 | 7.14 | 88.90 | 590.4 | 6 | 4.6 | -24.8 | -20.4 | -10.3 | 6.9 | 19.1 | 25.4 | 27.8 |
| 110101 | North | 664971 | 39.48 | 12.22 | 32.84 | 13699.3 | 8 | 13.4 | -7.0 | -4.0 | 2.5 | 14.7 | 24.1 | 28.4 | 30.3 |
| 120106 | North | 296822 | 39.05 | 11.89 | 43.66 | 24954.3 | 11 | 13.1 | -7.4 | -4.6 | 2.1 | 14.4 | 23.8 | 28.1 | 29.9 |
| 130227 | North | 876959 | 39.41 | 7.81 | 77.53 | 271.1 | 3 | 11.9 | -10.1 | -6.9 | 1.3 | 13.0 | 22.8 | 27.0 | 28.9 |
| 130721 | North | 693140 | 40.47 | 10.55 | 86.45 | 133.0 | 3 | 9.5 | -13.9 | -10.2 | -1.5 | 10.4 | 20.7 | 25.9 | 28.4 |
| 140107 | North | 1582398 | 37.47 | 10.14 | 47.76 | 15146.7 | 6 | 11.3 | -9.0 | -6.1 | 1.5 | 12.3 | 21.1 | 26.3 | 28.0 |
| 140427 | North | 815848 | 36.03 | 6.77 | 87.36 | 294.6 | 4 | 10.0 | -10.3 | -6.9 | 1.1 | 11.7 | 19.2 | 23.7 | 25.6 |
| 150103 | North | 541721 | 40.49 | 7.11 | 52.52 | 2254.6 | 3 | 8.1 | -16.4 | -12.6 | -2.9 | 9.4 | 19.7 | 25.3 | 27.8 |
| 150802 | North | 394555 | 41.34 | 7.09 | 69.09 | 18615.8 | 6 | 6.5 | -19.4 | -15.5 | -5.5 | 7.8 | 18.7 | 25.2 | 27.5 |
| 610202 | Northwest | 275003 | 35.49 | 12.01 | 58.78 | 1236.0 | 3 | 10.4 | -10.4 | -6.1 | 2.2 | 12.0 | 18.9 | 23.8 | 25.9 |
| 610326 | Northwest | 299555 | 34.15 | 8.44 | 79.67 | 347.6 | 4 | 13.6 | -4.8 | -2.0 | 5.1 | 14.6 | 21.9 | 27.5 | 29.8 |
| 610921 | Northwest | 558218 | 32.43 | 9.26 | 89.12 | 180.5 | 3 | 16.1 | 0.3 | 2.6 | 7.9 | 16.8 | 23.6 | 29.0 | 31.4 |
| 620702 | Northwest | 1343857 | 38.56 | 7.09 | 73.04 | 119.7 | 6 | 8.7 | -16.7 | -11.5 | -1.4 | 10.3 | 19.2 | 25.0 | 27.3 |
| 630103 | Northwest | 509880 | 36.43 | 9.61 | 55.41 | 1966.8 | 2 | 6.1 | -12.6 | -9.5 | -2.2 | 7.5 | 14.4 | 18.8 | 21.5 |
| 640104 | Northwest | 248779 | 38.28 | 6.81 | 53.08 | 894.9 | 4 | 10.3 | -13.5 | -9.1 | 0.5 | 12.1 | 20.7 | 26.1 | 27.9 |
| 640502 | Northwest | 1157120 | 37.32 | 6.24 | 79.78 | 63.9 | 4 | 9.9 | -14.2 | -8.9 | 0.5 | 11.6 | 19.9 | 25.1 | 26.8 |

**sTable 1. The characteristics for 66 communities included in this study (*continued*)**

| City code | Region | Population | Latitude  (degrees N) | Age^+^65  (%) | No High  School (%) | Pop. density  (person/km^2^) | daily average deaths | Temperature (℃) | | | | | | | |
| --- | --- | --- | --- | --- | --- | --- | --- | --- | --- | --- | --- | --- | --- | --- | --- |
|  |  |  |  |  |  |  |  | mean | 1% | 5% | 25% | 50% | 75% | 95% | 99% |
| 650102 | Northwest | 273506 | 43.47 | 7.99 | 44.48 | 4071.8 | 4 | 8.1 | -20.1 | -14.4 | -4.5 | 10.8 | 20.7 | 26.5 | 28.8 |
| 310103 | East | 594391 | 31.24 | 16.28 | 38.86 | 30904.2 | 6 | 17.5 | 0.4 | 2.5 | 9.7 | 18.6 | 25.1 | 30.6 | 32.6 |
| 310117 | East | 895957 | 31.24 | 6.07 | 62.16 | 2619.9 | 9 | 17.5 | 0.4 | 2.5 | 9.7 | 18.6 | 25.1 | 30.6 | 32.6 |
| 320111 | East | 1263289 | 31.56 | 7.89 | 54.46 | 778.0 | 8 | 16.6 | -1.7 | 0.7 | 8.3 | 17.7 | 24.9 | 30.0 | 32.0 |
| 320506 | East | 1282262 | 31.53 | 6.88 | 66.47 | 1559.5 | 9 | 16.8 | -1.3 | 0.9 | 8.7 | 17.9 | 25.0 | 30.2 | 32.3 |
| 320582 | East | 895582 | 32.05 | 9.69 | 71.22 | 1248.0 | 14 | 16.3 | -1.4 | 0.8 | 8.2 | 17.2 | 24.2 | 29.6 | 31.6 |
| 320831 | East | 575489 | 33.38 | 13.18 | 76.15 | 230.5 | 6 | 15.3 | -2.9 | -0.6 | 7.0 | 16.5 | 23.8 | 28.8 | 30.7 |
| 320921 | East | 944231 | 33.46 | 8.95 | 83.13 | 349.0 | 8 | 14.9 | -2.8 | -0.5 | 6.6 | 15.9 | 23.2 | 28.4 | 29.9 |
| 330103 | East | 871207 | 30.14 | 10.35 | 44.09 | 16722.7 | 5 | 17.8 | 0.5 | 2.6 | 10.1 | 18.7 | 25.7 | 31.2 | 32.9 |
| 330483 | East | 542873 | 30.36 | 10.51 | 83.08 | 1122.2 | 11 | 17.2 | -0.3 | 2.0 | 9.5 | 18.1 | 24.8 | 30.4 | 32.2 |
| 330523 | East | 1563050 | 30.14 | 10.1 | 81.75 | 247.4 | 7 | 17.8 | 0.5 | 2.6 | 10.1 | 18.7 | 25.7 | 31.2 | 32.9 |
| 340803 | East | 782255 | 30.32 | 10.89 | 61.01 | 1170.2 | 2 | 17.7 | -0.7 | 2.1 | 9.5 | 19.0 | 25.7 | 31.1 | 32.9 |
| 341823 | East | 671243 | 30.08 | 12.68 | 84.32 | 145.8 | 5 | 8.9 | -10.2 | -5.9 | 3.1 | 10.0 | 16.0 | 19.1 | 20.4 |
| 370203 | East | 526096 | 36.04 | 11.63 | 40.54 | 8722.2 | 9 | 13.2 | -5.0 | -2.0 | 5.1 | 14.3 | 21.6 | 25.8 | 27.6 |
| 370602 | East | 1246762 | 37.28 | 8.7 | 47.80 | 4770.4 | 8 | 13.1 | -5.6 | -2.9 | 4.6 | 14.4 | 21.9 | 26.0 | 28.1 |
| 370684 | East | 710298 | 37.38 | 12.48 | 77.22 | 399.9 | 8 | 13.3 | -5.1 | -3.0 | 4.0 | 14.5 | 22.9 | 27.1 | 28.9 |
| 370785 | East | 321251 | 36.45 | 10.97 | 80.30 | 586.9 | 13 | 13.3 | -6.4 | -3.8 | 3.8 | 14.5 | 23.1 | 27.6 | 29.4 |
| 360102 | Central | 656592 | 28.36 | 8.96 | 42.56 | 31361.8 | 4 | 18.7 | 0.3 | 3.6 | 10.8 | 20.1 | 26.5 | 31.6 | 33.2 |
| 360423 | Central | 377358 | 28.36 | 7.96 | 83.01 | 102.7 | 4 | 18.7 | 0.3 | 3.6 | 10.8 | 20.1 | 26.5 | 31.6 | 33.2 |
| 410526 | Central | 466552 | 36.03 | 8.08 | 87.55 | 709.3 | 18 | 13.9 | -5.4 | -3.1 | 4.4 | 15.3 | 23.4 | 28.3 | 30.4 |
| 411328 | Central | 360269 | 33.02 | 9.18 | 83.26 | 513.5 | 18 | 15.8 | -2.1 | 0.3 | 7.4 | 17.2 | 24.2 | 29.0 | 30.7 |
| 411502 | Central | 451109 | 32.08 | 8.4 | 64.39 | 333.4 | 7 | 16.0 | -2.5 | 0.1 | 8.1 | 17.1 | 24.0 | 29.1 | 31.2 |

**sTable 1. The characteristics for 66 communities included in this study (*continued*)**

| City code | Region | Population | Latitude  (degrees N) | Age^+^65  (%) | No High  School (%) | Pop. density  (person/km^2^) | daily average deaths | Temperature (℃) | | | | | | | |
| --- | --- | --- | --- | --- | --- | --- | --- | --- | --- | --- | --- | --- | --- | --- | --- |
|  |  |  |  |  |  |  |  | mean | 1% | 5% | 25% | 50% | 75% | 95% | 99% |
| 420102 | Central | 1050528 | 30.36 | 9.42 | 45.23 | 14450.9 | 11 | 17.6 | -0.9 | 2.1 | 9.3 | 18.8 | 25.8 | 31.3 | 33.1 |
| 420625 | Central | 830054 | 32.23 | 9.13 | 81.23 | 205.1 | 7 | 16.2 | -1.0 | 1.3 | 8.1 | 17.5 | 24.2 | 28.9 | 30.7 |
| 430181 | Central | 1192060 | 28.43 | 10.45 | 83.52 | 256.0 | 19 | 17.4 | -0.1 | 2.8 | 9.3 | 18.6 | 25.3 | 30.0 | 31.4 |
| 430626 | Central | 513746 | 28.43 | 9.96 | 79.20 | 229.7 | 14 | 17.4 | -0.1 | 2.8 | 9.3 | 18.6 | 25.3 | 30.0 | 31.4 |
| 500101 | Southwest | 947574 | 30.46 | 11.71 | 75.61 | 452.1 | 23 | 18.8 | 4.4 | 6.6 | 11.3 | 19.2 | 25.7 | 30.9 | 33.3 |
| 500225 | Southwest | 1157666 | 30.46 | 12.43 | 85.38 | 467.4 | 13 | 18.8 | 4.4 | 6.6 | 11.3 | 19.2 | 25.7 | 30.9 | 33.3 |
| 510105 | Southwest | 316179 | 30.45 | 10.05 | 38.76 | 12214.5 | 7 | 16.5 | 1.9 | 4.4 | 9.7 | 17.4 | 23.0 | 26.9 | 28.3 |
| 510182 | Southwest | 523607 | 31.01 | 11.47 | 81.42 | 536.9 | 13 | 16.0 | 1.2 | 4.0 | 9.2 | 16.7 | 22.5 | 26.6 | 28.2 |
| 511025 | Southwest | 762887 | 29.37 | 11.31 | 89.08 | 687.5 | 17 | 17.7 | 3.0 | 5.6 | 11.0 | 18.2 | 24.0 | 29.3 | 31.7 |
| 511325 | Southwest | 1279469 | 30.47 | 13.97 | 84.46 | 463.7 | 7 | 17.9 | 2.9 | 5.5 | 10.6 | 18.4 | 24.4 | 30.4 | 33.3 |
| 520302 | Southwest | 495129 | 27.46 | 7.51 | 73.33 | 1103.5 | 6 | 15.7 | -1.2 | 2.6 | 9.0 | 16.4 | 22.8 | 27.2 | 28.7 |
| 520328 | Southwest | 787449 | 27.46 | 9.88 | 88.73 | 202.9 | 5 | 15.7 | -1.2 | 2.6 | 9.0 | 16.4 | 22.8 | 27.2 | 28.7 |
| 530402 | Southwest | 299990 | 24.21 | 8.9 | 72.22 | 493.2 | 5 | 16.5 | 5.7 | 8.8 | 12.5 | 17.4 | 20.5 | 22.7 | 23.6 |
| 532627 | Southwest | 246147 | 24.04 | 6.51 | 93.43 | 100.8 | 10 | 17.4 | 2.4 | 5.8 | 13.1 | 19.0 | 22.4 | 24.9 | 26.8 |
| 540102 | Southwest | 828140 | 29.41 | 3.49 | 64.61 | 503.7 | 1 | 9.7 | -2.8 | -0.7 | 3.9 | 10.3 | 15.3 | 19.3 | 21.6 |
| 350521 | South | 200231 | 24.29 | 7.33 | 84.33 | 1950.9 | 11 | 21.2 | 8.3 | 10.7 | 16.1 | 21.8 | 26.7 | 29.2 | 30.3 |
| 440104 | South | 296987 | 23.11 | 11.48 | 38.59 | 34250.5 | 18 | 22.7 | 7.2 | 10.9 | 18.3 | 24.1 | 27.7 | 30.8 | 31.8 |
| 440282 | South | 279074 | 25.08 | 11.98 | 81.73 | 135.9 | 6 | 20.3 | 3.9 | 6.5 | 14.2 | 21.8 | 26.7 | 30.5 | 31.3 |
| 441284 | South | 678306 | 23.02 | 8.08 | 81.68 | 429.8 | 6 | 22.7 | 7.9 | 11.3 | 18.2 | 24.2 | 27.7 | 30.5 | 31.4 |
| 441424 | South | 507433 | 23.56 | 8.55 | 84.37 | 325.6 | 15 | 21.6 | 5.6 | 9.0 | 16.8 | 23.3 | 27.0 | 30.2 | 31.1 |
| 450126 | South | 378606 | 22.38 | 9.67 | 85.90 | 340.4 | 13 | 21.6 | 7.0 | 9.5 | 16.6 | 23.3 | 27.1 | 29.3 | 30.3 |
| 450521 | South | 696277 | 21.27 | 9.95 | 81.28 | 366.1 | 8 | 22.9 | 7.8 | 11.0 | 19.0 | 24.5 | 27.9 | 30.1 | 30.9 |

**
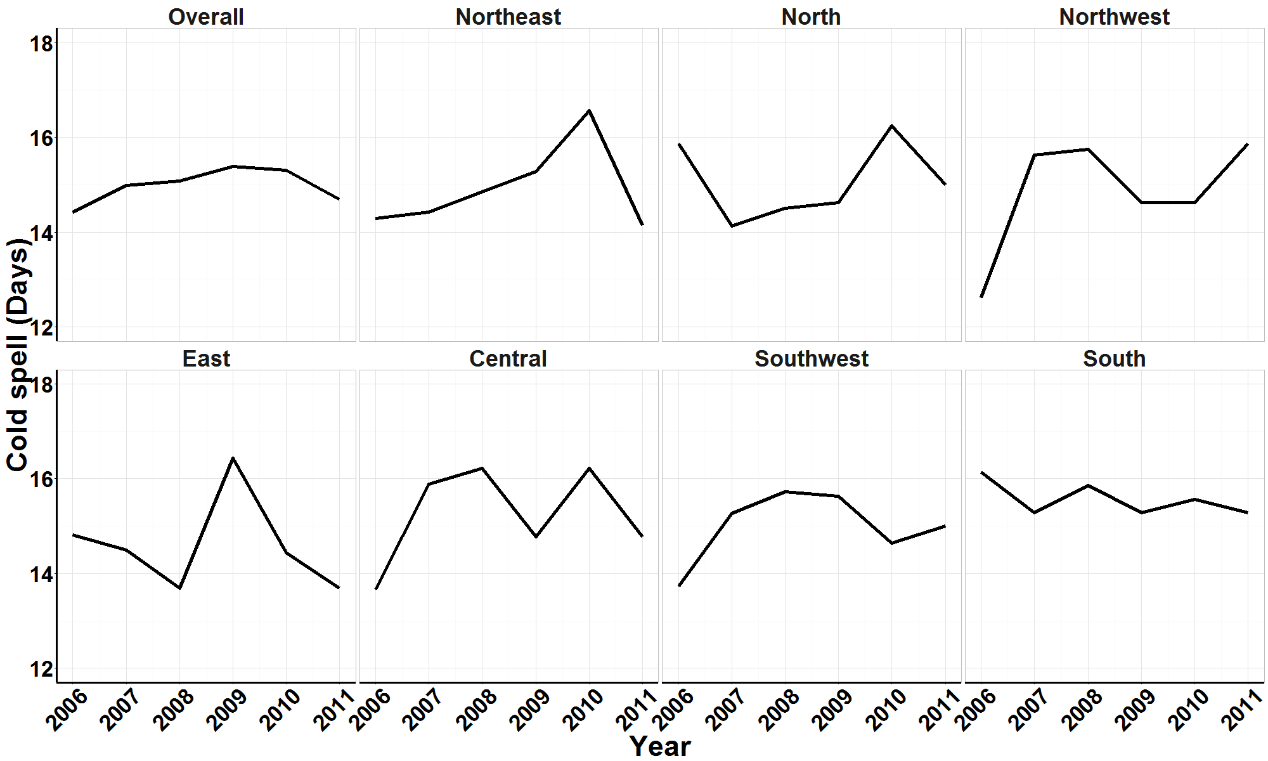
**

sFig 2. The temporal distribution of cold spells in different regions in China

**
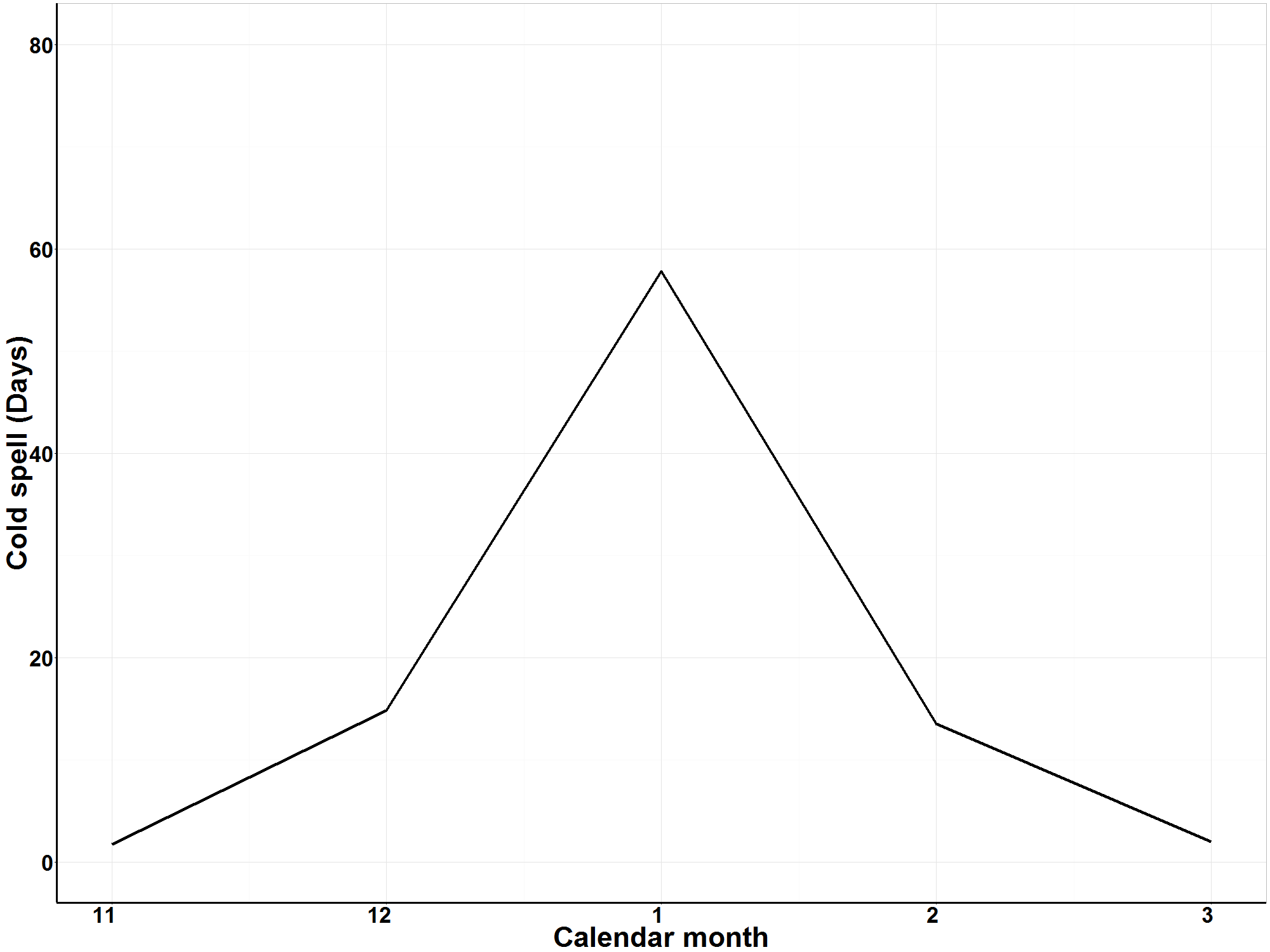
**

sFig 3. The monthly distribution of cold spells in all studied communities

sTable 2. Summary CER (95%CI) of different intensity of cold spells on total non-accidental mortality during lag 0-27 days in different regions of China, 2006-2011.

|  | 5^th^ percentile^†^ |  | 2.5^th^ percentile^‡^ |
| --- | --- | --- | --- |
|  | CER (%, 95%CI) |  | CER (%, 95%CI) |
| Overall | 28.2 (21.4, 35.3) |  | 39.7 (27.6, 52.9) |
| Northeast | 0.2 (-14.1, 16.8) |  | 2.3 (-14.8, 22.8) |
| North | 1.4 (-13.3, 18.5) |  | 9.5 (-16.9, 44.2) |
| Northwest | 17.7 (6.3, 30.3) |  | 21.8 (-5.7, 57.3) |
| East | 39.2 (26.5, 53.1) |  | 55.5 (36.0, 77.7) |
| Central | 36.8 (22.6, 52.6) |  | 57.1 (26.6, 94.8) |
| Southwest | 31.3 (19.9, 43.7) |  | 47.1 (27.8, 69.3) |
| South | 58.7 (40.1, 79.9) |  | 92.9 (54.9, 140.2) |

CER：Cumulative excess risk of mortality for cold spell exposure during lag 0-27 days.

†: a cold spell was defined as a weather fluctuation if the mean daily temperature fell below the 5th percentile of the study period (cold season in 2006-2011) in a specific community for at least 2 consecutive days.

‡: a cold spell was defined as a weather fluctuation if the mean daily temperature fell below the 2.5th percentile of the study period (cold season in 2006-2011) in a specific community for at least 2 consecutive days.


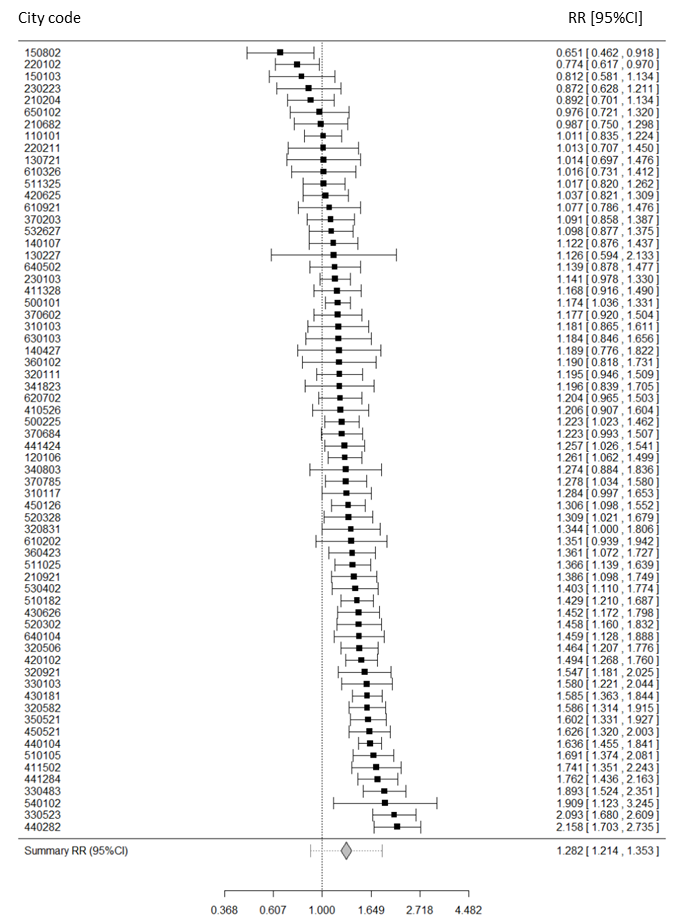


sFig 4. Summary RR (95% CI) of cold spells on total non-accidental mortality during lag 0-27 days in 66 communities, China, 2006-2011.

A cold spell was defined as a weather fluctuation if the mean daily temperature fell below the 5th percentile of the study period (cold season in 2006-2011) in a specific community for at least 2 consecutive days.

sTable 3. Characteristics of cold spell in 7 regions, China, 2006-2011.

|  | Northeast | North | Northwest | East | Central | Southwest | South |
| --- | --- | --- | --- | --- | --- | --- | --- |
| Daily number of deaths | 27.35 | 22.14 | 13.13 | 29.83 | 39.72 | 38.75 | 41.61 |
| Average daily TM (℃) | -17.42 | -9.43 | -8.83 | -1.56 | 0.27 | 3.33 | 8.38 |
| Number of cold spells | 137 | 180 | 173 | 244 | 218 | 241 | 188 |
| Cold spell characteristics |  |  |  |  |  |  |  |
| Intensity (average ℃) | -20.22 | -12.73 | -12.64 | -1.35 | -0.83 | 1.44 | 1.45 |
| Duration (average days (longest)) | 4.60(13) | 4.01(13) | 3.75 (12) | 2.53(11) | 3.65(16) | 3.68(15) | 3.05(14) |
| Timing (the first day in season)) | NOV 17^th^ | NOV 12^th^ | NOV 15^th^ | NOV 20^th^ | NOV 15^th^ | NOV 16^th^ | NOV 16^th^ |

NOV: November

sTable 4. Summary CER (95% CI) of cold spells with different intensity and duration on total non-accidental mortality during lag 0-27 days in different regions of China, 2006-2011.

|  | Cold spell duration | | | | |
| --- | --- | --- | --- | --- | --- |
|  | 2 days  CER (95%CI) |  | 3-5 days  CER (95%CI) |  | 6 days and more  CER (95%CI) |
| 5^th^ percentile^†^ |  |  |  |  |  |
| Overall | 18.9 (10.8, 27.7) |  | 31.9 (21.8, 42.9) |  | 36.3 (26, 47.4) |
| Northeast | -28.9 (-55.6, 13.9) |  | -0.8 (-21.5, 25.2) |  | 4.7 (-14.7, 28.5) |
| North | 5.6 (-29.2, 57.4) |  | 10.1 (-13.6, 40.2) |  | 7.7 (-16.7, 39.1) |
| Northwest | 18.9 (-48.5, 174.6) |  | 13.9 (-10.2, 44.4) |  | 32.3 (2.1, 71.5) |
| East | 2.2 (-16.8, 25.4) |  | 30.8 (14.2, 49.8) |  | 60 (42.6, 79.2) |
| Central | 26.3 (-15.3, 88.2) |  | 31.9 (11.7, 55.7) |  | 48.9 (23.0, 79.6) |
| Southwest | 60.1 (10.1, 132.9) |  | 36.7 (10.4, 69.2) |  | 29.3 (12.8, 48.2) |
| South | 56.5 (17.8, 108.0) |  | 45.8 (28.2, 65.9) |  | 81.9 (43.0, 131.5) |
| 2.5^th^ percentile^‡^ |  |  |  |  |  |
| Overall | 28.2 (13.0, 45.5) |  | 37.7 (22.6, 54.8) |  | 58.6 (27.9, 96.5) |
| Northeast | 5.6 (-12.8, 27.8) |  | 5.0 (-13.0, 26.8) |  | 0.8 (-23.3, 32.4) |
| North | 8.5 (-39.8, 95.5) |  | -12.2 (-46.5, 44) |  | -9.3 (-38.4, 33.7) |
| Northwest | -13.4 (-45.9, 38.6) |  | 2.0 (-36.0, 62.4) |  | 71.0 (-9.8, 224.5) |
| East | 64.8 (33.2, 104.0) |  | 57.1 (23.5, 99.7) |  | 36.4 (-22.5, 140.3) |
| Central | 15.6 (-11.7, 51.2) |  | 81.1 (51.6, 112.3) |  | 86.1 (-9.9, 284.5) |
| Southwest | 25.3 (-10.8, 76.0) |  | 32.7 (3.6, 70.1) |  | 114.8 (49.5, 208.7) |
| South | 43.8 (2.1, 102.6) |  | 92.9 (57.5, 136.3) |  | 138 (50.9, 275.4) |

CER：Cumulative excess risk of mortality for cold spell exposure during lag 0-27 days.

†: A cold spell was defined as a weather fluctuation if the mean daily temperature fell below the 5th percentile of the study period (cold season in 2006-2011) in a specific community for at least 2 consecutive days.

‡: A cold spell was defined as a weather fluctuation if the mean daily temperature fell below the 2.5th percentile of the study period (cold season in 2006-2011) in a specific community for at least 2 consecutive days.

Under each definition of a cold spell, the duration of cold spells were divided into three groups: short duration (lasting 2 days), moderate duration (lasting 3 to 5 days) and long duration (lasting 6 days or more).

sTable 5. Summary CER (95% CI) of average 10 days earlier occurrence of cold spells on non-accidental mortality during lag 0-27 days in different regions of China, 2006-2011.

|  | Cold spell timing (every 10 days earlier) | | | | |
| --- | --- | --- | --- | --- | --- |
|  | 2 days  CER (95%CI) |  | 3-5 days  CER (95%CI) |  | 6 days and more  CER (95%CI) |
| 5^th^ percentile^†^ |  |  |  |  |  |
| Overall | 1.9 (0.5, 3.4) |  | 1.4 (0.8, 2.0) |  | 2.4 (1.6, 3.3) |
| Northeast | -2.1 (-5.1, 1.1) |  | -0.4 (-1.8, 1.1) |  | 0.5 (-0.7, 1.7) |
| North | 1.2 (-5.5, 8.3) |  | 0 (-2.1, 2.1) |  | -3.1 (-7, 0.9) |
| Northwest | 0.1 (-4.8, 5.2) |  | 0.7 (-1.6, 3.2) |  | 1.3 (0, 2.7) |
| East | -0.4 (-1.9, 1.2) |  | 1.9 (0.8, 2.9) |  | 4.9 (3.5, 6.2) |
| Central | 2.3 (-1.3, 6.0) |  | 1.2 (-0.6, 3.1) |  | 3.8 (1.2, 6.5) |
| Southwest | 6.2 (0.8, 11.9) |  | 2.2 (0.7, 3.7) |  | 2.3 (1.2, 3.3) |
| South | 5.9 (3.2, 8.7) |  | 2.9 (1.6, 4.2) |  | 7.6 (4.4, 11.0) |
| 2.5^th^ percentile^‡^ |  |  |  |  |  |
| Overall | 1.8 (-0.8, 4.4) |  | 2.3 (0.8, 3.9) |  | 7.3 (0.6, 14.5) |
| Northeast | -0.5 (-4.5, 3.8) |  | 0.5 (-1.8, 2.9) |  | 0.7 (-3.5, 5) |
| North | 1.3 (-8.1, 11.6) |  | -2.8 (-8.7, 3.4) |  | - |
| Northwest | 1.2 (-7.8, 11.0) |  | 1 (-3.3, 5.4) |  | 3.5 (-0.9, 8.1) |
| East | 5.4 (2.3, 8.6) |  | 4.1 (-0.3, 8.7) |  | 6.4 (-2.5, 16) |
| Central | 0.0 (-7.3, 7.9) |  | 5.5 (2.7, 8.5) |  | 11.3 (2.6, 20.8) |
| Southwest | 6.9 (-0.8, 15.2) |  | 1.9 (-1, 4.9) |  | 6.5 (3.8, 9.4) |
| South | 4.2 (1.2, 7.3) |  | 4.5 (-0.5, 9.7) |  | - |

CER：Cumulative excess risk of mortality for cold spell exposure during lag 0-27 days.

†: A cold spell was defined as a weather fluctuation if the mean daily temperature fell below the 5th percentile of the study period (cold season in 2006-2011) in a specific community for at least 2 consecutive days.

‡: A cold spell was defined as a weather fluctuation if the mean daily temperature fell below the 2.5th percentile of the study period (cold season in 2006-2011) in a specific community for at least 2 consecutive days.

Under each definition of a cold spell, the duration of cold spells were divided into three groups: short duration (lasting 2 days), moderate duration (lasting 3 to 5 days) and long duration (lasting 6 days or more).

-: In the north and south communities, the effects were not estimated because there were very few cold spells lasting 6 days or more and the regression models were not successfully fit.

sTable 6. Summary CER (95% CI) of cold spells with different duration on total non-accidental mortality during lag 0-27 days in China, by age, gender, education, and causes of death

|  | Cold spell timing (every 10 days earlier) | | | | |
| --- | --- | --- | --- | --- | --- |
|  | 2 days  CER (95%CI) |  | 3-5 days  CER (95%CI) |  | 6 days and more  CER (95%CI) |
| Causes of death |  |  |  |  |  |
| Overall | 18.9 (10.8, 27.7) |  | 31.9 (21.8, 42.9) |  | 36.3 (26, 47.4) |
| CVD | 21.9 (12.5, 32.1) |  | 33.7 (22, 46.4) |  | 35.4 (23.4, 48.5) |
| CBD | 15 (2.6, 28.8) |  | 25.9 (12.4, 41) |  | 36.7 (24.2, 50.6) |
| RESP | 37 (20.1, 56.2) |  | 48.5 (35.4, 62.9) |  | 69.2 (49.2, 91.7) |
| Age of death (years) |  |  |  |  |  |
| 0-64 | 12.1 (2.1, 23) |  | 18.1 (7.3, 30) |  | 14.9 (4.3, 26.5) |
| 65-74 | 10.4 (1.4, 20.3) |  | 22 (12, 33) |  | 37.7 (25.5, 51.1) |
| 75-84 | 9.6 (0.5, 19.4) |  | 20.1 (10.2, 30.9) |  | 22 (12, 33) |
| ≥85 | 33 (20.2, 47.2) |  | 49.5 (33.3, 67.7) |  | 52.5 (33.6, 74.2) |
| Gender |  |  |  |  |  |
| Males | 22.1 (13.9, 30.8) |  | 34.9 (25.2, 45.3) |  | 29.8 (18.9, 41.7) |
| Females | 13.1 (4.3, 22.7) |  | 24.7 (13.1, 37.4) |  | 42.4 (30.6, 55.3) |
| Education |  |  |  |  |  |
| Primary | 4.3 (-7.2, 17.1) |  | 18.3 (7, 30.8) |  | 45.3 (28.1, 64.8) |
| Middle school | 15 (-4, 37.7) |  | 26.7 (6, 51.4) |  | 3.2 (-11.6, 20.5) |
| Collage or over | -5.2 (-29.8, 28.1) |  | -2.7 (-22.5, 22.2) |  | -5.5 (-23.6, 16.9) |

CER：Cumulative excess risk of mortality for cold spell exposure during lag 0-27 days.

A cold spell in this figure was defined as a weather fluctuation if the mean daily temperature fell below the 5th percentile of the study period (cold season in 2006-2011) in a specific community for at least 2 consecutive days.

The duration of cold spells were divided into three groups: short duration (lasting 2 days), moderate duration (lasting 3 to 5 days) and long duration (lasting 6 days or more).

**sTable 7**. Sensitivity analyses on the effects of *df* per year/month on the associations between cold spell^a^ and total non-accidental mortality during lag 0-27 days of China, 2006-2011

|  | *Df* | Overall | |
| --- | --- | --- | --- |
|  |  | CER (%) | 95%CI |
| Per year | 2 | 29.7 | 22.8 to 37.0 |
|  | 3 | 28.2 | 21.4 to 35.3 |
|  | 4 | 27.4 | 20.7 to 34.5 |
| Per month | 2 | 28.2 | 21.5 to 35.4 |
|  | 3 | 28.2 | 21.4 to 35.3 |
|  | 4 | 26.6 | 19.9 to 33.6 |

^a^A cold spell is defined by the daily mean temperature falling below the 5^th^ percentile of the study period, community-specific distribution for at least 2 consecutive days.
